# Aligning Motivation, Expectations and Pedagogy: A Behavioural Science Framework for Widening Access Mature Students (WAMSs) in Higher Education

**DOI:** 10.3390/bs16071105

**Published:** 2026-07-03

**Authors:** Nazim Uddin

**Affiliations:** Quality Assurance and Enhancement Management Committee, Nelson College London, 406-410 Eastern Avenue, Ilford IG2 6NQ, UK; n.uddin@nelsoncollege.ac.uk

**Keywords:** widening participation, mature students, adult learning, self-determination theory, expectancy–value theory, cognitive load theory, persistence, institutional design, motivation, higher education

## Abstract

Widening participation has expanded access to higher education, yet disparities in retention and completion among mature students persist, indicating limits in existing structural and engagement-based explanations. This study addresses this gap by developing Behavioural Alignment Theory (BAT), a framework that conceptualises persistence as an emergent outcome of alignment between motivational regulation, expectancy recalibration and instructional architecture. Using a conceptual integration and theoretical mapping methodology, the article synthesises Self-Determination Theory, Expectancy–Value Theory and Cognitive Load Theory, grounded in foundational and contemporary literature on mature and widening access students. The analysis shows that persistence is shaped through dynamic interaction between institutional design and behavioural processes. Evidence indicates that misalignment across these domains can destabilise engagement, while coherent alignment supports sustained participation under conditions of role complexity and constraint. The study concludes that persistence is not reducible to individual attributes or isolated institutional factors, but emerges from system-level interaction between psychological processes and institutional conditions. The contribution of BAT lies not in the invention of new constructs, but in providing a mechanism-explicit mid-range integrative framework that specifies how established constructs interact within institutional settings to shape persistence or withdrawal.

## 1. Introduction

Over the past three decades, widening participation has become a defining policy objective across higher education systems in advanced economies, underpinned by commitments to social mobility, economic competitiveness and lifelong learning (Baker et al., 2025; Gale & Parker, 2014). Mature students, those entering higher education through delayed, interrupted or non-linear pathways, are increasingly positioned as central actors within this transformation, particularly in the context of reskilling and workforce transition agendas. However, while participation has expanded, outcomes have not equalised. Persistent disparities in retention, progression and completion among widening access mature students remain evident in higher education (Thomas, 2012; Yorke & Longden, 2008). Hayman et al. (2024) report that mature students are “more likely to drop out,” achieve poorer degree outcomes, and contend with greater family, financial, caring and work commitments, while many experience a socially, emotionally and financially challenging first year. Yet dropout is still too often framed either as an individual deficit or as a simple failure of integration.

Recent national data in England, for example, indicate that mature and part-time participation has declined over time and remains uneven, where mature learners continue to be treated as a priority group within access and participation policy (Office for Students, 2018, 2025). At the same time, attrition remains unevenly distributed. Evidence across multiple national contexts suggests that withdrawal is disproportionately associated with factors such as financial pressure, employment commitments, caregiving responsibilities and weak institutional integration, rather than purely academic ability (Christie et al., 2005; Zepke, 2017).

This empirical pattern reveals a central paradox: while widening participation has expanded access, it has been less successful in securing equitable persistence. Entry represents a structural achievement; persistence, by contrast, unfolds as a temporally extended behavioural process shaped by the interaction between institutional conditions and student decision-making under constraint. Emerging evidence suggests that, for adult learners, academic variables may exert a stronger influence on dropout than social integration (Lee, 2025), calling into question the continued reliance on integration models developed around traditional student populations. For mature students—whose participation is structured by time scarcity, geographical distance and competing responsibilities—such models risk theoretical misfit. More broadly, the literature increasingly conceptualises dropout as a multidimensional process rather than a single-cause problem, shaped by interacting economic, academic and psychosocial factors (Barragán Moreno & González Támara, 2024; Jerez, 2024; Kahu & Nelson, 2018). Taken together, these findings suggest that persistence among mature learners is best understood not as a function of isolated variables, but as an ongoing negotiation between structural constraint, psychological processes and the perceived feasibility of continued participation.

Existing scholarship offers a theoretically sophisticated account of inequality at the structural level. Bourdieusian analyses of capital and habitus demonstrate how institutional norms privilege dominant cultural forms and reproduce advantage (Bourdieu, 1986; Reay, 2018), while integration models position academic and social engagement as central to persistence (Tinto, 1993; Kahu & Nelson, 2018). More recent syntheses reinforce the importance of belonging, showing robust associations with “academic engagement, motivation, persistence, and overall achievement,” yet also reveal persistent conceptual indeterminacy in how such constructs are defined and operationalised (Allen et al., 2024). However, these accounts remain less precise in specifying how structural positioning, institutional experiences and educational interactions are translated into day-to-day persistence behaviour.

Kahu and Nelson (2018) make a significant contribution by locating student engagement within the educational interface, where student characteristics and institutional practices interact through psychosocial mechanisms such as self-efficacy, emotions, belonging and well-being. Trowler et al. (2022) extend this position by reconceptualising engagement as reciprocal, dynamic and multidimensional, distinguishing engagement of and by students and foregrounding critical, political and socio-cultural dimensions. Together, these frameworks move student engagement theory beyond individualised and deficit accounts by showing that persistence is shaped through situated student–institution interaction.

Behavioural Alignment Theory (BAT) advances these accounts by shifting the focus from engagement broadly conceived to the behavioural mechanisms of mature student persistence. BAT specifies how institutional conditions become consequential through the interaction of motivational regulation, expectancy recalibration and instructional architecture. Its contribution lies not in adding further engagement constructs, but in integrating SDT, EVT and CLT to explain how autonomy support, success signalling, perceived cost and cognitive manageability align or misalign over time. In doing so, BAT reframes widening participation as a problem of behavioural alignment between institutional design and the motivational, evaluative and cognitive conditions required for sustained participation.

At the same time, emerging evidence from adult and mature learner populations challenges the generalisability of these frameworks, indicating that academic conditions and external constraints may exert greater influence on persistence than social integration alone (Hayman et al., 2024; Lee, 2025). Parallel critiques further problematise dominant framings, with resilience discourse shown to “prioritise individual agency” in ways that risk obscuring structural inequalities (Ellis & Johnston, 2024). Systematic reviews similarly converge on the conclusion that dropout is irreducibly multidimensional, shaped by interacting academic, socioeconomic and institutional factors rather than discrete predictors (Sánchez et al., 2023). Collectively, this body of work provides a compelling account of how inequality is structured and reproduced. However, it remains analytically limited in specifying how persistence is behaviourally enacted over time. In particular, it offers insufficient precision in modelling the dynamic regulation of motivation, the formation and recalibration of expectations, and the ways in which institutional conditions structure sustained participation.

This article intervenes directly in this analytical gap by advancing a behavioural science perspective that moves beyond structural explanation to process specification. It argues that widening participation requires not only structural access but psychological access, that is, the alignment of institutional conditions with the motivational, cognitive and evaluative processes through which engagement is sustained over time. Within this framing, persistence is conceptualised not as the product of discrete variables but as an emergent outcome of interaction across three interdependent domains: motivational regulation, expectancy recalibration and instructional architecture. By integrating Self-Determination Theory (SDT), Expectancy–Value Theory (EVT) and Cognitive Load Theory (CLT), the article develops Behavioural Alignment Theory (BAT) as a mechanism-explicit mid-range framework that specifies how institutional design conditions behavioural trajectories. In doing so, it does not claim originality through entirely new constructs; instead, its contribution lies in a theoretically integrated account capable of linking macro-level structures to micro-level processes of persistence.

## 2. Materials and Methods

### 2.1. Methodological Orientation

This study adopts a conceptual integration and theoretical mapping approach to advance theory-building rather than data generation. Its purpose is to develop a mechanism-explicit framework capable of explaining mature student persistence as a dynamic behavioural process. This aligns with contemporary approaches to conceptual research, where theory development proceeds through systematic synthesis, problematisation and integration of existing knowledge to enhance explanatory precision (Jaakkola, 2020; MacInnis, 2011; Post et al., 2020).

Widening participation research remains fragmented because its major traditions explain different dimensions of the problem without being fully integrated into a shared mechanism-level account. Sociological work explains how inequality is structured through capital, habitus, class, institutional hierarchy and stratification; educational research examines how engagement, belonging, transition and support shape student experience; and psychological research explains motivation, expectancy, self-efficacy and cognitive load. Each domain is theoretically valuable, but they often operate at different levels of analysis, using different concepts, methods and outcome measures. Conceptual integration is therefore appropriate because dropout and persistence are increasingly recognised as multidimensional phenomena shaped by academic, socioeconomic, institutional and psychological factors, while the mechanisms linking these factors remain under-specified. The fragmentation is therefore not evidence of theoretical weakness, but of partial explanation across separate domains.

Behavioural Alignment Theory addresses this gap by reconstructing these insights into a mechanism-explicit framework that explains how institutional design jointly shapes motivation, expectations and cognitive sustainability, rather than treating them as isolated predictors. The methodological stance is conceptual rather than empirical, but it remains empirically grounded. The article does not generate primary data; instead, it systematically engages with established and recent evidence to identify underlying mechanisms, integrate fragmented insights and develop a coherent theoretical framework for explaining mature student persistence.

### 2.2. Literature Scope and Analytical Strategy

The literature was selected through purposive theoretical sampling rather than exhaustive systematic review procedures, prioritising sources with the greatest conceptual relevance and explanatory leverage. An exhaustive systematic review would not have served the purpose of this article because the aim is not to measure the weight of evidence on a single empirical question. The aim is to build a mid-range theory by selecting sources that explain different parts of the persistence problem and then mapping how these explanations can be connected. Sources were selected to capture both foundational theoretical traditions and contemporary evidence relevant to mature, adult, part-time and post-traditional learners. The analysis follows four stages designed to ensure conceptual clarity, analytical parsimony and explanatory coherence.

#### 2.2.1. Stage 1: Reconstruction of Widening Participation Theory

This stage critically reconstructs widening participation scholarship by engaging foundational and contemporary literature, including capital theory (Bourdieu, 1986; Reay, 2018), integration models (Tinto, 1975, 1993; Kahu & Nelson, 2018) and stratification perspectives (Marginson, 2016; Boliver, 2015). Recent work on belonging, identity and post-traditional participation is also incorporated (Allen et al., 2024; Gale & Parker, 2014; Hayman et al., 2024). The aim is analytical clarification, identifying explanatory strengths alongside conceptual ambiguity and under-theorised processes. This reveals a consistent pattern of structural sophistication coupled with behavioural under-specification.

#### 2.2.2. Stage 2: Theoretical Mapping Across Behavioural Domains

Three complementary behavioural frameworks are mapped to distinct domains:

- Self-Determination Theory (SDT): Motivational regulation (Ryan & Deci, 2017).
- Expectancy–Value Theory (EVT): Evaluation of success, value and cost (Eccles & Wigfield, 2002).
- Cognitive Load Theory (CLT): Cognitive processing and instructional design (Sweller, 2011).

These frameworks are selected for their empirical robustness and explanatory complementarity. SDT, EVT and CLT were selected because they address complementary dimensions of mature student persistence: motivational regulation, expectancy appraisal, and cognitive sustainability. Together, they explain why students continue to invest effort, how they evaluate the feasibility of success, and whether learning remains manageable under conditions of adult role complexity. These theories were therefore selected not because they exhaust all explanations of persistence, but because together they specify the behavioural mechanisms most directly implicated in mature students’ continuation decisions: motivation, feasibility appraisal and cognitive manageability. Each is treated as analytically distinct to avoid conceptual overlap: SDT addresses regulatory quality, EVT captures evaluative judgement, and CLT explains cognitive sustainability. This differentiation ensures conceptual precision and prevents theoretical conflation.

#### 2.2.3. Stage 3: Cross-Theoretical Synthesis

The third stage identifies interaction effects across these three domains. Rather than treating motivation, expectations and pedagogy as additive variables, the analysis conceptualises them as interdependent processes operating within a dynamic system. The expectation of non-linearity arises from the possibility of threshold effects and reinforcing feedback processes. Small changes in expectancy, perceived cost or cognitive burden may have limited consequences until critical thresholds are reached, at which point engagement and continuation decisions can shift rapidly. Recent behavioural and educational research emphasises such interactional and context-dependent dynamics (Kahu & Nelson, 2018; Lee, 2025; Uddin, 2025). The synthesis identifies mechanism-level pathways between the three domains. Instructional architecture shapes the cognitive conditions under which mastery experiences become attainable; poorly structured learning environments increase cognitive burden and reduce opportunities to experience competence. BAT distinguishes competence as a psychological need from competence-related task experience. Within SDT, competence refers to the felt experience of effectiveness and mastery, whereas within CLT, instructional conditions determine whether such mastery experiences are cognitively achievable. Cognitive overload is therefore not equivalent to competence; rather, it constrains the conditions through which competence can be experienced. Experiences of competence and autonomy, in turn, influence expectancy because students who feel capable and self-endorsed are more likely to judge success as attainable. Perceived cost affects both motivational regulation and cognitive sustainability, as time pressure, financial strain and role conflict reduce willingness to invest effort while simultaneously constraining cognitive resources. These processes are mutually reinforcing: excessive cognitive burden can undermine competence experiences, weakened competence can depress expectancy, and high perceived cost can amplify both motivational and cognitive strain. Persistence is therefore conceptualised as an emergent outcome of alignment across cognitive sustainability, motivational regulation and expectancy appraisal, rather than as the result of any single factor.

#### 2.2.4. Stage 4: Formalisation of Behavioural Alignment Theory (BAT)

The final stage specifies BAT as a coherent and testable framework through:

- Construct definition (motivation, expectancy, instructional architecture).
- Boundary conditions (e.g., mature learners, high role complexity).
- Mechanism-explicit pathways (e.g., expectancy collapse, competence threat, autonomy frustration).
- Propositional development linking constructs to persistence outcomes.

This formalisation ensures analytical precision and empirical tractability.

### 2.3. Conceptual Control and Epistemological Positioning

Conceptual integration carries two principal risks: conceptual inflation and theoretical conflation (Suddaby, 2010). Conceptual inflation occurs when a framework expands excessively by incorporating too many constructs, thereby weakening explanatory precision and making the theory difficult to operationalise. Theoretical conflation occurs when distinct concepts or theories are merged without preserving their analytical boundaries, producing ambiguity about what each construct explains. This study mitigates these risks in three ways. First, it uses domain differentiation: SDT is restricted to motivational regulation, EVT to expectancy, value and cost appraisal, and CLT to cognitive sustainability. Second, it adopts non-additive integration, treating the theories as interacting mechanisms rather than interchangeable or cumulative variables. Third, it applies analytical parsimony, including only constructs that are theoretically necessary and empirically grounded. In this way, BAT preserves conceptual clarity while enabling integration across domains without unnecessary expansion or theoretical overlap.

The study adopts a relational epistemology in which behaviour emerges from interaction between institutional structures and psychological processes. This position rejects both structural determinism and individual reductionism, aligning with contemporary work that emphasises the co-constitution of institutional conditions and student experience (Kahu & Nelson, 2018; Allen et al., 2024). Behavioural processes are therefore understood as institutionally mediated rather than individually isolated.

### 2.4. Empirical Grounding and Limitations

Although no primary data are collected, the framework is grounded in empirical research. It draws on established studies of mature student participation and persistence (Christie et al., 2005; Zepke, 2017), as well as recent work on adult learners, engagement and institutional alignment (Hayman et al., 2024; Lee, 2025; Lyu et al., 2025). It also incorporates emerging WAMS-focused research on motivation, pedagogy and learning design (Uddin et al., 2023; Uddin et al., 2026; Hasan et al., 2025). This ensures that theoretical development reflects observed behavioural patterns rather than abstract speculation.

The primary output is a mid-range theoretical framework that specifies mechanisms linking institutional design to persistence outcomes. As a mid-range framework, BAT does not claim to explain all forms of student success, nor is it confined to a single institutional case; rather, it offers a bounded, testable account of how institutional conditions are translated into persistence through identifiable behavioural mechanisms. It integrates previously distinct theoretical traditions and generates propositions suitable for longitudinal and multi-level empirical testing. The framework is intended to inform both research and institutional practice by identifying how pedagogical and organisational design conditions shape motivation, expectations and cognitive sustainability, thereby influencing sustained engagement over time.

A further limitation is that BAT is developed primarily from the literature on mature and widening access learners, with some empirical illustrations drawn from specific institutional and national contexts. The contextual work cited throughout the article includes a number of studies authored by the present author and collaborators. These studies are included because of their direct relevance to mature learner motivation, pedagogy and engagement, but they should be regarded as contextually informative rather than the primary evidential basis for BAT, which is grounded in broader interdisciplinary literature. Independent empirical testing is therefore required to establish the robustness and transferability of the framework. Consequently, the framework should be regarded as a theoretically informed account of persistence whose broader applicability across higher education systems, disciplines and student populations requires further empirical testing.

As a conceptual study, causal claims remain theoretically inferred and require empirical validation. Similarly, BAT’s proposition that persistence may exhibit non-linear dynamics remains theoretical rather than empirically demonstrated in the present article and should be interpreted as a testable proposition. The integration of multiple frameworks involves interpretive judgement, and the model is initially developed within the context of mature learners. However, these limitations are inherent to theory-building and are offset by the framework’s conceptual clarity, scope and testability.

## 3. Empirical Foundations and Evidence Base for Behavioural Alignment

Although Behavioural Alignment Theory (BAT) is developed conceptually, it is grounded in substantial and expanding empirical literature on widening access mature students (WAMSs). This literature spans motivation, engagement, pedagogy and institutional design, but remains fragmented across domains. Studies typically examine discrete variables such as motivation, belonging or attainment, with limited attention to their interaction.

The purpose of this section is to synthesise key empirical findings and reinterpret them through a mechanism-oriented lens. In doing so, it demonstrates that the core domains of BAT, namely motivational regulation, expectancy recalibration and instructional architecture, are already empirically observable but remain theoretically unintegrated.

### 3.1. Motivational Profiles: Instrumental Entry and Internalisation

A consistent finding across widening participation research is the predominance of instrumentally oriented participation among mature learners. Entry into higher education is typically motivated by career advancement, financial improvement and professional transition rather than intrinsic interest in subject matter (Baker et al., 2025; Uddin et al., 2026). Empirical and review-based research confirms the centrality of future-oriented and utility-based considerations in shaping engagement decisions, particularly where participation is undertaken alongside employment and caregiving responsibilities (Eccles & Wigfield, 2002; Wigfield & Cambria, 2010). However, recent research also emphasises that motivation is multidimensional, with learners combining instrumental goals with aspirations for personal development, identity reconstruction and social mobility (Kahu & Nelson, 2018; Urhahne & Wijnia, 2023).

This motivational profile has important implications for persistence. While instrumental motives provide a strong basis for entry, they are often insufficient to sustain engagement under conditions of constraint. Contemporary research demonstrates that externally oriented motivation remains comparatively fragile unless it becomes internalised through experiences that support autonomy and competence (Ryan & Deci, 2017). Empirical evidence indicates that such need-supportive environments are associated with stronger engagement and persistence, whereas controlled forms of motivation are more vulnerable to disruption (Zepke, 2017; Hayman et al., 2024). Studies of adult learners similarly show that persistence is strengthened when learning is perceived as relevant to life goals and aligned with prior experience, while externally regulated motivation is more susceptible to competing demands (Kasworm, 2010; Hayman et al., 2024).

Longitudinal research reinforces this dynamic perspective by demonstrating that persistence depends less on initial motivation than on its transformation over time. Motivation evolves as learners interpret institutional experiences, with trajectories shaped by feedback, perceived progress and the alignment between study and identity (Kahu et al., 2020; Tempelaar et al., 2015). More recent studies further indicate that motivation is mediated by contextual factors such as self-regulation, social interaction and perceived competence, which directly influence engagement and continuation (Lee, 2025; Lyu et al., 2025). Where students are able to connect their studies to a coherent sense of purpose, motivation stabilises; where such alignment is weak or disrupted, motivation may decline even when initial commitment is strong.

Motivation is therefore not a fixed attribute that students bring into higher education, but a dynamic and context-dependent process continually shaped by institutional conditions. Persistence depends not only on why students enter, but on how their motivations are reinforced, recalibrated or undermined over time through their interaction with the learning environment.

### 3.2. Expectancy Recalibration: Institutional Signalling and Confidence

Expectancy beliefs are a central determinant of persistence because students continue when they believe that success is both attainable and worthwhile. Expectancy–Value Theory argues that engagement depends on the interaction between perceived likelihood of success, subjective task value and perceived cost (Eccles & Wigfield, 2002). For mature students, this calculus is particularly consequential. Many enter higher education with strong extrinsic and utility-oriented motivations, including career advancement, financial improvement and professional transformation. However, high value does not automatically produce persistence. Where opportunity costs are substantial, including time scarcity, lost earnings, caregiving responsibilities and psychological strain, students must continually reassess whether continued participation remains feasible.

For mature learners, expectancy is often uncertain at entry because of educational discontinuity, unfamiliar academic conventions and limited recent experience of assessment. These initial beliefs are therefore highly sensitive to institutional signals. Assessment clarity, feedback quality, curriculum relevance and communication play a decisive role in recalibrating students’ judgements about whether they can succeed. Early assessment experiences are especially influential because they provide concrete evidence about standards, progress and the possibility of improvement. Transparent feedback can strengthen perceived competence and stabilise expectancy, whereas ambiguous criteria, inconsistent marking or delayed feedback may rapidly undermine confidence (Yorke & Longden, 2008; Christie et al., 2005).

Recent longitudinal and learning analytics research further suggests that expectancy trajectories matter for continuation. Students whose expectations stabilise or improve are more likely to persist, while declining expectancy is associated with withdrawal risk (Tempelaar et al., 2015). In this sense, persistence is shaped not only by students’ initial motivation or ability, but by how institutions structure and signal success over time. For mature students, expectancy recalibration is therefore inseparable from value and cost: high utility value may sustain effort only when institutional design keeps success credible and participation manageable.

### 3.3. Instructional Design: Cognitive Sustainability

Instructional design is a critical but often under-recognised determinant of persistence, particularly for mature and post-traditional learners. Cognitive Load Theory demonstrates that learning depends on the relationship between task complexity, instructional structure and the limits of working memory (Sweller, 2011; Sweller et al., 2011). For mature students, cognitive sustainability is especially salient because study is undertaken alongside employment, caregiving and other competing demands, increasing baseline cognitive and emotional load. Under these conditions, the design of learning environments becomes central to whether engagement is experienced as manageable or overwhelming.

Empirical research indicates that poorly structured curricula, unclear instructions, fragmented delivery and misaligned technologies increase extraneous cognitive load, leading to confusion, reduced perceived competence and disengagement (Kirschner et al., 2006; Paas et al., 2003). More recent studies further show that excessive task demands relative to learner resources undermine self-regulation and increase the likelihood of disengagement, whereas well-calibrated instructional design supports sustained effort and persistence (de Bruin et al., 2020; Sweller et al., 2019). Conversely, structured and scaffolded learning environments reduce unnecessary processing demands and enable students to focus on meaningful learning, thereby strengthening confidence and engagement (Chi et al., 1982; Sweller et al., 2011). For returning learners, early mastery experiences remain particularly important in rebuilding academic confidence following educational discontinuity (Kasworm, 2010).

Recent work on contemporary learning environments reinforces the importance of coherent instructional architecture. Studies show that effective design—characterised by clear sequencing, integrated feedback and alignment between tasks and outcomes—supports cognitive manageability and sustained engagement, while fragmented systems increase cognitive burden and undermine learning efficiency (Mayer, 2020; Fiorella & Mayer, 2016). Evidence from mature student research similarly highlights the importance of clarity, relevance and pedagogical coherence, particularly where learning must align with adult identity and real-world application (Hasan et al., 2025; Uddin et al., 2023). Digital and blended provision can enhance flexibility and access, but only where systems are stable and pedagogically integrated; otherwise, they increase fragmentation, reduce perceived control and weaken engagement (Papé et al., 2025). Instructional design therefore functions as behavioural infrastructure, shaping cognitive sustainability, perceived competence and motivational stability, and ultimately influencing whether continued participation is experienced as feasible and worthwhile.

### 3.4. Relational Pedagogy and Recognition

Relational pedagogy and identity integration are not treated as separate BAT domains because they do not perform distinct explanatory functions outside the three core mechanisms. Rather, they operate as cross-domain mechanisms through which institutional conditions shape motivational regulation, expectancy recalibration and instructional architecture. Relational pedagogy influences autonomy, relatedness, confidence and interpretive clarity, while identity integration connects learning to prior experience, future goals and perceived relevance. They are therefore central to the empirical foundations of BAT, but analytically they work through the three core domains rather than expanding the framework.

Under the conditions under which mature students enter higher education, tutor–student relationships become behaviourally consequential: they signal whether students are recognised as legitimate participants, whether their prior experience is valued, and whether institutional expectations are navigable. Therefore, relational pedagogy is necessary within the empirical foundations of Behavioural Alignment Theory because mature student persistence is not shaped only by motivation, assessment or instructional design, but also by the quality of recognition students experience within the learning environment. Empirical studies of mature and widening access students consistently show that clarity, respect, responsiveness, fairness and recognition of prior experience are highly valued because they support confidence, belonging and sustained engagement (Hayman et al., 2024; Kahu & Nelson, 2018; Uddin et al., 2023; Zepke, 2017).

Relational pedagogy is therefore a mechanism through which persistence is stabilised. Autonomy-supportive teaching strengthens students’ sense of agency, competence-supportive feedback enhances confidence, and respectful adult-to-adult interaction fosters relatedness and legitimacy (Deci & Ryan, 2000; Ryan & Deci, 2017). Review evidence on belonging in higher education shows that feeling accepted, valued and connected is strongly associated with engagement, motivation, persistence and achievement (Allen et al., 2024; Strayhorn, 2019; Thomas, 2012). For mature learners, however, belonging is often less dependent on traditional campus socialisation and more dependent on being treated as capable adults whose life experience is recognised as an epistemic resource. Where such recognition is absent, students may experience identity misalignment, reduced confidence and weakening commitment, even where instrumental motivation remains strong.

Relational pedagogy also interacts with the other domains of behavioural alignment. Clear and respectful communication helps students interpret expectations, reduces uncertainty and strengthens confidence in their capacity to succeed. Timely and constructive feedback can stabilise expectancy by showing that improvement is possible, while pedagogical relevance links learning to adult identity and future goals (Hasan et al., 2025; Jerez, 2024; Lyu et al., 2025). Conversely, dismissive, impersonal or opaque interactions may increase psychological cost, undermine competence and make continued participation appear less viable. Relational pedagogy is therefore central to mature student persistence because it connects institutional design with motivational regulation, expectancy stability and cognitive manageability. It justifies inclusion in the empirical foundations because it explains how institutional relationships become behavioural mechanisms rather than merely background conditions.

### 3.5. Life Experience and Identity Integration

Mature learners bring substantial life, work and caregiving experience into higher education, and this experience should be understood not as a compensatory background characteristic but as an epistemic resource. Empirical studies show that mature students actively draw on prior experience to interpret concepts, test abstract knowledge against practice and make learning personally meaningful (Hasan et al., 2025; Kasworm, 2010; Uddin, 2023). Recent work similarly indicates that mature students’ transition into higher education is shaped by identity renegotiation, prior educational experience and the extent to which institutional environments recognise adult learner identities (Hayman et al., 2024; Jones & McConnell, 2023).

Pedagogical approaches that emphasise relevance, reflection and application are therefore central to persistence because they enable mature learners to connect academic knowledge with professional identity, life history and future aspirations. Case-based, experiential and problem-centred learning can strengthen engagement by allowing students to move inductively between lived experience and academic theory, thereby making learning cognitively accessible and identity-relevant (Hasan et al., 2025; Uddin, 2023). Empirical studies of mature student experience also show that collaborative learning, peer relationships and recognition of adult responsibilities contribute to successful engagement, particularly where students are balancing study with caring or employment commitments (Hayman et al., 2024; Homer, 2022).

Persistence is therefore linked not only to academic performance but to the perceived coherence between learning and identity. Where higher education validates life experience as a legitimate basis for knowledge construction, mature learners are more likely to experience study as purposeful, confidence-building and professionally transformative. Conversely, where curricula disregard prior experience or treat mature students as deficient entrants into a traditional model, identity misalignment may weaken engagement even when initial motivation remains strong. Life experience and identity integration are therefore essential to Behavioural Alignment because they explain how relevance-oriented pedagogy converts participation into sustained commitment.

### 3.6. Cost, Constraint and Dropout Risk

Cost remains a central determinant of persistence, particularly for mature and post-traditional learners. Within the expectancy–value tradition, cost encompasses time demands, financial burden and psychological strain, all of which shape the feasibility of continued participation (Eccles & Wigfield, 2002). For mature students, these costs are structurally elevated due to employment, caregiving responsibilities and opportunity costs associated with foregone income or time. Empirical research consistently shows that such constraints are central to participation decisions, with mature learners disproportionately affected by financial pressures, time scarcity and competing responsibilities (Christie et al., 2005; Lee, 2025; Sánchez et al., 2023).

Evidence further indicates that withdrawal is more often the result of cumulative external pressures than a lack of motivation or ability. Large-scale and longitudinal studies demonstrate that dropout risk increases when high perceived cost interacts with low expectancy of success and adverse study conditions, leading students to reassess the viability of continued participation (Tempelaar et al., 2015; Barragán Moreno & González Támara, 2024). Studies of adult learners similarly show that external constraints, rather than academic inability, are primary drivers of discontinuation, particularly where institutional demands conflict with work and family responsibilities (Zepke, 2017; Lee, 2025).

Institutional support plays a critical moderating role in this process. Empirical evidence shows that flexible scheduling, clear assessment structures, timely feedback and accessible support services can reduce perceived cost by making participation more manageable and predictable (Yorke & Longden, 2008; Kahu & Nelson, 2018; Lyu et al., 2025). Research on mature student transitions further demonstrates that structured support and responsive teaching can buffer the effects of external pressures by enhancing students’ sense of control and feasibility (Hayman et al., 2024). In this sense, cost is not solely an external constraint but is partially shaped by institutional design. Persistence is therefore contingent on the balance between external demands and institutional support, where effective organisational and pedagogical conditions can mitigate constraint and sustain engagement under pressure.

### 3.7. Synthesis: Empirical Basis for Behavioural Alignment

The empirical literature reviewed across the preceding analysis converges on a central proposition: student persistence is not reducible to individual capability or isolated predictors but emerges from the alignment between behavioural practices and institutional conditions. Evidence from engagement, motivation, instructional design, relational pedagogy and life-context constraints collectively indicates that persistence is an emergent, system-level outcome shaped by the interaction between how students act and how institutions structure those actions. Behavioural alignment therefore functions as an integrative construct that connects micro-level practices of engagement with macro-level institutional design.

Across these domains, a consistent pattern is the dynamic and processual nature of alignment. Students do not enter higher education fully aligned; rather, they continuously recalibrate their behaviours in response to institutional signals such as assessment practices, feedback, pedagogical structure and relational interactions. Motivation evolves through internalisation, expectancy shifts through performance cues, and cognitive engagement adjusts in response to instructional clarity and workload demands. Persistence is therefore less a function of initial conditions than of whether these processes stabilise over time. Where students are able to synchronise effort, expectations and cognitive resources with institutional demands, engagement becomes sustainable. Where such synchronisation fails, disengagement becomes increasingly likely.

However, the empirical evidence also reveals a structural asymmetry. While institutions implicitly require behavioural alignment, they frequently fail to make expectations sufficiently transparent, accessible or responsive to diverse learner profiles, producing conditions in which misalignment is systematically generated rather than individually chosen (Hayman et al., 2024; Kahu & Nelson, 2018; Lyu et al., 2025). Mature and widening access students are particularly affected, as their participation is shaped by external constraints, discontinuous educational histories and non-traditional identities. Under such conditions, misalignment is often misrecognised as individual deficit rather than as a consequence of opaque assessment, excessive cognitive demand or limited recognition of prior experience. Behavioural alignment must therefore be understood as a reciprocal process, requiring institutional adaptation as much as student adjustment.

The synthesis further demonstrates that alignment operates through the interaction of distinct but interdependent mechanisms. Motivational stability depends on the internalisation of purpose and relevance; expectancy depends on credible signals of progress and success; cognitive sustainability depends on manageable instructional design; relational pedagogy shapes confidence, legitimacy and belonging; and external cost conditions constrain the feasibility of continued participation. These processes do not operate independently. Excessive cognitive demand can undermine perceived competence; unclear assessment can destabilise expectations; high external cost can erode motivation even where value remains high. Persistence emerges when these domains are mutually reinforcing and breaks down when misalignment in one domain propagates across others.

At a theoretical level, the evidence suggests that existing frameworks capture important dimensions of persistence but remain analytically partial: they illuminate specific aspects of the student experience, yet do not provide a fully integrated account of how structural conditions, institutional design and behavioural mechanisms interact over time to produce persistence or withdrawal. Sociological accounts explain structural inequality but do not specify how it becomes behaviourally consequential in real time. Educational and engagement-based models identify correlates of participation but often lack precision in specifying mechanisms. Psychological theories offer detailed accounts of motivation and cognition but are frequently detached from institutional design. Behavioural alignment integrates these perspectives by specifying how institutional conditions shape behavioural processes and how these processes, in turn, produce persistence outcomes.

Notwithstanding its explanatory value, the empirical base remains constrained. Much of the literature relies on cross-sectional designs and self-reported measures, thereby limiting insight into the temporal dynamics of alignment and restricting understanding of how it evolves over time, including how it is progressively strengthened, destabilised or broken down. Conceptual inconsistency further complicates synthesis, as engagement, belonging, motivation and persistence are often used interchangeably without clear analytical distinction. In addition, the literature tends to privilege institutional success metrics such as retention and completion, with less attention to students’ lived experience of alignment or to the possibility that partial or strategic misalignment may represent adaptive responses to constraint (Christie et al., 2005; Hayman et al., 2024; Kahu & Nelson, 2018; Zepke, 2017). Finally, the predominance of research in Western higher education systems limits the generalisability of findings, suggesting that alignment processes may be contextually contingent.

Taken together, the empirical evidence supports the central claim that persistence is a relational and dynamic process shaped by the degree of coherence between institutional design and behavioural mechanisms. Behavioural alignment is therefore not an individual attribute but an emergent outcome of system-level interaction, comprising individual inputs, institutional processes and interactions, and resulting in outcomes such as improved retention and student success. Advancing this field requires greater conceptual precision, stronger longitudinal and multi-level methodologies, and a more critical examination of how institutional practices structure the conditions under which alignment becomes possible.

## 4. Discussion: Behavioural Alignment Theory and System-Level Explanation

### 4.1. From Integration to Specification

Building on the preceding theoretical and empirical synthesis, this section formalises Behavioural Alignment Theory (BAT) as a mechanism-explicit framework of mature student persistence. The central move is to specify how motivation, expectations and instructional conditions interact over time within institutional contexts to produce sustained engagement or withdrawal.

BAT conceptualises persistence as an emergent outcome of institutional–psychological coherence rather than the additive effect of independent variables. BAT argues that mature students are more likely to persist when the institution makes study feel worthwhile, possible and manageable. Study feels worthwhile when motivation is supported. It feels possible when students receive clear signals that success is achievable. It feels manageable when teaching, assessment and feedback reduce unnecessary cognitive burden. When these three conditions align, persistence becomes more likely. When they do not, withdrawal risk increases. This shifts analysis from linear models towards a non-linear, interactional account in which outcomes depend on the alignment of interdependent behavioural processes. In doing so, the framework addresses a central limitation in widening participation research, namely the absence of an integrated account linking structural conditions to behavioural dynamics over time.

### 4.2. Conceptual Challenges and Safeguards

The development of Behavioural Alignment Theory raises several conceptual and methodological challenges. The first concerns theoretical conflation. Because BAT integrates Self-Determination Theory, Expectancy–Value Theory and Cognitive Load Theory, there is a risk that these theories may be merged without preserving their distinct explanatory boundary. This risk is addressed by assigning each theory a distinct role: SDT explains motivational regulation, EVT explains judgements of success, value and cost, and CLT explains the cognitive manageability of learning.

A second challenge is conceptual inflation. BAT could become overly broad if it absorbs every relevant factor associated with student persistence. To avoid this, the framework retains only three core domains. Relational pedagogy, belonging and identity are not treated as additional domains; they operate through the three core mechanisms. This keeps BAT focused and prevents it from becoming a general theory of student success.

A third challenge concerns causal overreach. As a conceptual theory-building article, BAT cannot claim to demonstrate causal pathways empirically. Its mechanisms are therefore presented as theoretically inferred propositions rather than demonstrated causal effects. This positioning opens the framework to longitudinal, mixed-methods and multi-level testing.

A fourth challenge is psychological reductionism. Because BAT draws on behavioural and psychological theories, there is a risk that persistence may be interpreted as a function of individual motivation, cognition or resilience. BAT explicitly resists this reading by framing persistence as institutional–psychological. Motivation, expectancy and cognitive load are treated as processes shaped by institutional design, assessment practices, pedagogy, feedback and support structures.

A fifth challenge concerns normative alignment. The concept of alignment may imply that students should adapt to existing institutional norms. This would reproduce the deficit logic that widening participation research has long criticised. BAT therefore defines alignment as reciprocal: institutions must adapt their institutional design, rather than expecting students alone to conform to dominant institutional expectations.

A sixth challenge is boundary ambiguity. BAT is expected to have the greatest explanatory power where participation is characterised by high role complexity, elevated perceived cost, educational discontinuity, and substantial institutional variability. These conditions are particularly characteristic of mature, widening access and post-traditional learners. The framework therefore advances a contextually bounded mid-range theory.

Finally, BAT faces a measurement challenge. Constructs such as motivational regulation, expectancy recalibration and cognitive load are context-sensitive and difficult to operationalise consistently. This does not invalidate the framework, but it requires careful empirical design.

Together, these safeguards clarify that BAT is not an unbounded synthesis or a deterministic model. It is a mid-range framework that integrates distinct theoretical domains while preserving conceptual boundaries, specifying scope conditions and generating propositions for empirical testing.

### 4.3. Core Domains

Having clarified these safeguards, BAT can now be specified through its three core domains: motivational regulation, expectancy recalibration and instructional architecture. These domains are interdependent, but they are not interchangeable. Each explains a different aspect of mature student persistence. Together, they explain why students continue, whether success still appears possible, and whether learning remains manageable.

#### 4.3.1. Motivational Regulation

Motivational regulation refers to the quality and stability of students’ reasons for persistence. This domain is grounded in Self-Determination Theory and is concerned with the internalisation of motives through autonomy, competence and relatedness support (Ryan & Deci, 2017).

For mature students, motivational regulation is especially important because entry into higher education is often instrumentally oriented, linked to career progression, financial improvement or professional transition (Kasworm, 2010; Uddin et al., 2026). These motives may be strong at entry, but they remain unstable unless they become integrated with adult identity, life goals and a developing sense of professional self. Pedagogical practices contribute to this process when they affirm agency, respect prior experience, provide meaningful rationale and support learners’ sense of capability (Hayman et al., 2024; Ryan & Deci, 2017; Uddin, 2023).

The boundary of this domain is therefore motivational rather than evaluative or cognitive. Motivational regulation concerns why students continue to invest themselves in study and whether that investment feels volitional and legitimate. It is weakened when institutional interactions are experienced as controlling, dismissive or infantilising, because such conditions frustrate autonomy and undermine internalisation (Christie et al., 2005; Zepke, 2017).

Although analytically distinct, motivational regulation remains interdependent with the other two domains. This boundary prevents conceptual inflation: motivational regulation is a precise construct explaining the internalisation and sustainability of mature students’ reasons for participation.

#### 4.3.2. Expectancy Recalibration

Expectancy recalibration refers to the ongoing updating of students’ beliefs about their likelihood of success, the value of participation and the associated costs, as shaped by their interaction with institutional signals. Grounded in Expectancy–Value Theory, this domain is concerned with evaluative judgement: how students interpret evidence about performance, standards and progress over time (Eccles & Wigfield, 2002; Wigfield & Cambria, 2010).

For mature learners, expectancy is often initially uncertain because of educational discontinuity, unfamiliarity with contemporary academic practices and limited prior exposure to assessment standards in higher education (Christie et al., 2005; Hayman et al., 2024). This uncertainty reflects a lack of calibrated reference points for judging what constitutes success and how performance is evaluated. As a result, expectancy is particularly sensitive in early stages of study and is shaped through iterative interaction with institutional signals.

BAT conceptualises expectancy as a temporal and dynamic process, characterised by early volatility, followed by potential stabilisation and either consolidation or decline. Students continuously interpret assessment outcomes, feedback, grading criteria and communication to infer whether they are meeting expectations, whether improvement is possible and whether continued investment is justified (Yorke & Longden, 2008; Tempelaar et al., 2015).

Expectancy recalibration explains how students judge the viability of success over time, based on institutional evidence. It is strengthened when assessment is transparent, feedback is timely and consistent, and expectations are clearly communicated. Conversely, ambiguity, inconsistent standards and weak signalling can destabilise expectancy, leading to declining confidence and increased withdrawal risk (Kahu & Nelson, 2018; Barragán Moreno & González Támara, 2024).

Expectancy recalibration is therefore analytically distinct: it explains how students evaluate, update and stabilise their beliefs about continuation within higher education.

#### 4.3.3. Instructional Architecture

Instructional architecture refers to the design and organisation of the learning environment, including curriculum structure, sequencing of tasks, assessment design, feedback systems and modes of delivery. Grounded in Cognitive Load Theory, this domain is concerned with how instructional conditions shape the cognitive demands placed on learners, particularly in terms of intrinsic, extraneous and germane load (Sweller, 2011; Sweller et al., 2019).

For mature students, instructional architecture is particularly consequential because learning takes place under conditions of heightened time pressure, competing responsibilities and limited cognitive bandwidth (Christie et al., 2005; Lee, 2025). Under such conditions, poorly structured curricula, fragmented delivery and unclear task requirements can increase extraneous load, leading to confusion, reduced efficiency and diminished perceptions of capability (Kirschner et al., 2006; Paas et al., 2003). Empirical studies show that excessive or poorly coordinated demands can overwhelm learners’ cognitive resources, making sustained engagement difficult even where motivation remains high.

Conversely, coherent and scaffolded instructional design can support cognitive sustainability, enabling students to manage complexity and maintain engagement over time. Structured sequencing, aligned assessment, clear guidance and integrated feedback reduce unnecessary processing demands and allow learners to focus on meaningful learning (Sweller et al., 2019; Mayer, 2020). Evidence from mature student research further indicates that clarity, relevance and well-integrated delivery modes are associated with stronger engagement and persistence, particularly where learning must be balanced with external commitments (Hasan et al., 2025; Papé et al., 2025).

The boundary of this domain is therefore cognitive and structural. Instructional architecture explains how the design of learning environments enables or constrains cognitive processing.

Instructional architecture thus functions as behavioural infrastructure, shaping the conditions under which other processes operate. Where design is coherent and manageable, it supports sustained engagement; where it is fragmented or excessively demanding, it can destabilise both confidence and participation. This conceptual boundary ensures precision: instructional architecture is not a general account of teaching quality or engagement, but a specific mechanism through which institutional design structures the cognitive feasibility of persistence.

### 4.4. Mature Students Persistence

Mature students’ persistence is best understood as an emergent outcome of the dynamic alignment between motivational regulation, expectancy recalibration and instructional architecture, rather than as the product of any single domain. These three elements operate as analytically distinct but interdependent processes that, when aligned, produce institutional–psychological coherence and sustained engagement over time.

Motivational regulation shapes the extent to which engagement is internally endorsed and identity-congruent. However, this motivational commitment is continuously tested and recalibrated through expectancy processes, as students interpret signals about their likelihood of success, the value of continued participation and the costs involved. At the same time, both motivation and expectancy are conditioned by instructional architecture, which determines whether learning is cognitively manageable, coherently structured and feasible alongside competing life demands. Persistence therefore does not depend solely on strong motivation, positive expectations or effective teaching in isolation, but on the mutual reinforcement of these processes.

Where alignment is achieved, these domains stabilise one another. Autonomy-supportive and relevant pedagogy strengthens internalised motivation; clear and consistent assessment and feedback stabilise expectations; and well-structured learning environments reduce cognitive burden and support perceived competence. Under such conditions, students are more likely to experience engagement as purposeful, achievable and sustainable. This produces a reinforcing cycle in which motivation, confidence and capability co-evolve, enabling persistence even under conditions of external constraint.

Conversely, misalignment in any domain can destabilise the system. For example, strong initial motivation may be undermined if assessment is opaque and expectancy declines, or if poorly structured learning environments generate excessive cognitive load. Similarly, clear expectations may not sustain engagement if motivation remains externally regulated or if instructional demands exceed available cognitive resources. These disruptions are not isolated; they propagate across domains, leading to declining confidence, increased perceived cost and eventual withdrawal.

BAT therefore proposes that persistence may be non-linear, especially where role complexity, high cost and institutional uncertainty interact. It emerges when motivational, evaluative and cognitive conditions are coherently aligned, and becomes fragile when this alignment is disrupted. Mature students’ success is thus not simply a function of individual effort or structural access, but of the extent to which institutional design enables the sustained integration of motivation, expectations and cognitive engagement over time.

### 4.5. Behavioural Alignment Dynamics: Interaction, Alignment, Boundary Conditions and Propositions

**Figure 1:**
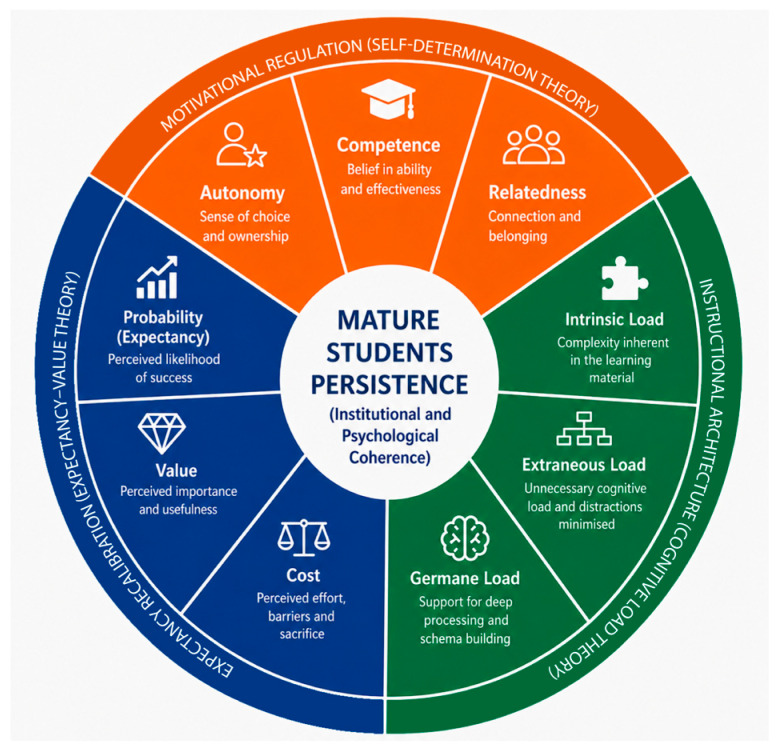
Behavioural Alignment Model.

As illustrated in the model (Figure 1), BAT proposes a systemic account in which persistence may display non-linear features, in which mature student persistence emerges from the interaction of motivational regulation, expectancy recalibration and instructional architecture. Persistence is therefore unlikely to be fully explained as an additive accumulation of independent effects, but a relational outcome contingent on institutional–psychological coherence across domains. This implies threshold effects, where acute expectancy decline or excessive cognitive overload can destabilise engagement despite otherwise favourable conditions; asymmetry, where negative conditions exert disproportionately stronger effects than positive conditions can offset; and dynamic feedback, where processes reinforce or weaken one another over time. For instance, declining expectancy may reduce engagement, impair performance and further erode confidence, whereas coherent pedagogical design can strengthen perceived competence, stabilise expectations and support motivational internalisation. A threshold is crossed when disruption in one domain exceeds the compensatory capacity of the others. For example, strong motivation may sustain engagement while assessment remains difficult but intelligible; however, if feedback is opaque, perceived cost is high and cognitive burden becomes excessive, expectancy may fall below the point at which continued participation appears feasible. BAT proposes that non-linearity may arise not from any single adverse condition, but from the accumulation of misalignment across domains until students reassess persistence as no longer viable. At the same time, the model recognises limitations: expectancy and motivation are subject to individual subjectivity and prior experience, while instructional design effects may vary depending on disciplinary context and instructor practice.

Within this system, persistence depends on mechanisms of alignment and misalignment. Alignment occurs when institutional conditions produce coherence across domains. These processes are unlikely to work as simple additive factors. BAT proposes that they interact: strength in one domain may support another, while serious weakness in one domain may reduce the effect of the others. Withdrawal is therefore not the result of a single deficit but the cumulative consequence of systemic misalignment, although the model, as illustrated in Figure 1, does not assume deterministic collapse, recognising that partial compensation and temporary persistence may still occur.

The applicability of BAT is bounded by specific contextual conditions. It is most relevant where participation is characterised by high role complexity, elevated perceived cost, educational discontinuity and institutional variability. These conditions are typical of widening access and mature students, whose engagement is shaped by employment, caregiving responsibilities and non-linear educational trajectories.

Consistent with this framework, BAT generates a set of testable propositions linking institutional design to behavioural outcomes. Autonomy-supportive pedagogy is expected to enhance motivational internalisation and persistence; transparent assessment and timely feedback should stabilise expectancy and reduce withdrawal risk; and excessive extraneous cognitive load is likely to undermine perceived competence and engagement. Expectancy decline is expected to mediate the relationship between institutional opacity and withdrawal, while motivational internalisation moderates the impact of perceived cost on continuation. Overall, persistence should be strongest where motivation, expectancy and instructional conditions are aligned as a coherent system. These propositions render BAT amenable to longitudinal, multi-level and mixed-methods empirical testing, while preserving its non-linear and interactional logic.

The behavioural alignment model (Figure 1) is a simplified representation designed to make complex relationships analytically visible. It captures the framework’s core architecture and principal interaction pathways, while the full complexity of contextual contingencies, feedback processes and temporal dynamics is developed in the accompanying discussion.

### 4.6. From Conceptual Integration to Mechanism-Explicit Explanation

This article advances widening participation research by integrating structural and behavioural perspectives into a mechanism-explicit account of persistence. Rather than displacing structural explanation, it incorporates it, specifying how institutional conditions are translated into behavioural processes over time. Behavioural Alignment Theory (BAT) therefore contributes by moving beyond descriptive or variable-based accounts of dropout towards an explanation of how persistence unfolds through the interaction of motivational regulation, expectancy recalibration and instructional architecture within institutional contexts.

Widening participation scholarship has generated a rich and influential body of work across sociological, educational and policy traditions (Reay, 2018; Gale & Parker, 2014; Kahu & Nelson, 2018). However, as recent reviews indicate, the field remains comparatively under-specified at the level of behavioural mechanism, often privileging structural diagnosis over processual explanation (Tight, 2020; Zepke, 2021). BAT intervenes at this point by retaining the explanatory strengths of structural accounts while specifying the micro-level processes through which persistence is enacted, stabilised or disrupted.

### 4.7. From Variable-Based Models to Non-Linear Systems

A primary contribution of BAT lies in shifting analysis from variable-based explanation to interactional, non-linear modelling. Existing research has identified key correlates of persistence, including engagement, belonging and motivation, but these are frequently treated as independent predictors (Thomas, 2012; Kahu & Nelson, 2018). While empirically valuable, such approaches obscure the dynamic interdependence through which these processes operate over time.

BAT conceptualises persistence as a system-level outcome characterised by feedback, threshold effects and asymmetry. Motivation, expectancy and instructional architecture are not additive influences but interdependent mechanisms. Motivation shapes the quality of engagement, expectancy structures judgements of viability, and instructional architecture conditions cognitive feasibility. Their interaction explains why similar students experience divergent outcomes across institutional contexts and why improvements in one domain do not necessarily translate into persistence when misalignment persists elsewhere. This aligns with broader developments in behavioural science that emphasise complexity, context sensitivity and feedback processes (Byrne & Callaghan, 2013; Michie et al., 2011).

### 4.8. Extending Sociological and Educational Accounts

BAT complements rather than replaces established theoretical traditions. Sociological frameworks, particularly Bourdieusian analyses, provide powerful accounts of how inequality is structured through capital, habitus and institutional fields (Bourdieu, 1986; Reay, 2018), yet they offer limited specification of how these conditions become behaviourally consequential in real time. BAT addresses this by identifying the mechanisms through which structural positioning shapes persistence, including the effects of misrecognition on expectancy, perceived competence and perceived cost.

Similarly, integration and engagement theories highlight the importance of academic and social participation (Tinto, 1993, 2017; Kahu & Nelson, 2018), but do not fully specify how engagement is produced, stabilised or disrupted. BAT extends these perspectives by articulating the interdependent processes through which engagement becomes sustainable or fragile. In this sense, BAT operates as a bridging framework, linking macro-level structural conditions with micro-level behavioural dynamics without reducing inequality to individual disposition.

BAT’s explanatory surplus lies in specifying how student–institution interaction becomes behaviourally consequential. BAT extends engagement interface theory in one specific way. Kahu and Nelson (2018) explain where student–institution interaction occurs: the educational interface. Trowler et al. (2022) show that engagement is reciprocal, dynamic and multidimensional. BAT builds on both, but asks a narrower behavioural question: how do institutional experiences become continuation or withdrawal decisions over time?

BAT specifies the mechanism sequence through which institutional design affects persistence: pedagogy and assessment shape cognitive manageability; cognitive manageability influences competence experience; competence and autonomy shape expectancy; and expectancy, value and cost appraisals inform continuation decisions. BAT therefore moves the discussion from the conditions of engagement to the behavioural mechanisms of persistence.

### 4.9. Reframing Widening Participation

A further contribution lies in reframing widening participation from a problem of access to one of behavioural alignment. While policy has traditionally prioritised entry, evidence demonstrates that access alone does not ensure equitable outcomes (Thomas, 2012; Zepke, 2017). BAT shifts analytical focus towards the conditions required to sustain engagement over time.

This reconceptualisation has three implications. First, it foregrounds persistence rather than entry as the critical outcome. Second, it challenges deficit narratives by locating barriers within institutional design rather than individual characteristics. Third, it emphasises the role of designable conditions, suggesting that persistence can be shaped through the alignment of pedagogy, assessment and support systems. This aligns with broader calls to adopt more holistic understandings of student success, encompassing belonging, wellbeing and meaningful participation (Kahu et al., 2020; Zepke, 2021).

### 4.10. Generalisability and Post-Traditional Higher Education

Although BAT is developed in relation to mature students, it may also apply to other learners who study under similar conditions, including role complexity, high participation costs, educational discontinuity and institutional variation. These conditions are becoming more common as higher education systems become more flexible, lifelong and diverse (Marginson, 2016; OECD, 2022).

BAT’s applicability is therefore expected to depend less on student age than on the presence of role complexity, participation costs, educational discontinuity and institutional variation. Whether the framework operates similarly in more traditional higher education contexts remains an empirical question. BAT is likely to offer less explanatory value where external role complexity and participation costs are limited.

### 4.11. Conceptual Integration: Avoiding Conflation and Inflation

The integration of multiple theoretical traditions introduces risks of conflation and conceptual inflation. BAT addresses these through structured integration, maintaining clear analytical boundaries between Self-Determination Theory, Expectancy–Value Theory and Cognitive Load Theory. Each contributes a distinct explanatory function: motivational regulation explains internalisation, expectancy recalibration explains evaluative judgement, and instructional architecture explains cognitive feasibility (Ryan & Deci, 2020; Eccles & Wigfield, 2020; Sweller et al., 2019).

By integrating these at the level of interaction rather than substitution, BAT preserves conceptual clarity while producing a coherent multidimensional account. This represents a methodological contribution by demonstrating how behavioural theories can be combined without loss of analytical precision.

### 4.12. Contribution to and Implications for Behavioural Science

BAT contributes to behavioural science by reinforcing the proposition that behaviour is contextually structured and dynamically produced. Motivation is not a stable trait but evolves through interaction with institutional environments; expectations are shaped by signals of success and progress; and cognitive capacity is influenced by instructional design. These insights align with emerging approaches that conceptualise behaviour as the outcome of person–environment interactions rather than isolated psychological variables (Michie et al., 2011; Walton & Crum, 2020).

By extending these principles into higher education, BAT provides a concrete model, Figure 1, of how institutional design shapes behavioural outcomes over time.

More specifically, BAT contributes to behavioural science by explaining how behaviour is shaped under conditions of constraint, how continuation decisions evolve through ongoing appraisal of success, value and cost, and how institutional design influences motivation, cognitive manageability and persistence through reinforcing feedback processes. In doing so, it advances behavioural science beyond individual-level explanations by demonstrating how organisational and educational environments function as behavioural infrastructure that systematically shapes behavioural outcomes. The article further extends behavioural science into higher education by integrating motivational, evaluative and cognitive mechanisms within a single framework, thereby providing a mechanism-based account of how institutional conditions influence persistence over time.

### 4.13. Future Research Trajectories

BAT establishes a foundation for a programme of empirical research focused on the temporal dynamics of persistence. Key directions include examining how institutional signalling shapes expectancy trajectories, how relevance-oriented pedagogy supports motivational internalisation, how learning environments function as collaborative social spaces, and how autonomy support interacts with economic constraint.

Addressing these questions requires longitudinal and multi-level research designs capable of capturing interaction effects and feedback processes over time (Kahu et al., 2020; Tight, 2020). Such approaches are essential for testing the causal pathways and boundary conditions specified by the framework.

Nevertheless, BAT should be understood as a conceptual framework rather than an empirically validated model. Its integration of SDT, EVT and CLT involves interpretive judgement, and the causal pathways proposed remain theoretically inferred from existing evidence rather than directly demonstrated. Furthermore, the framework’s assumptions regarding threshold effects, feedback dynamics and non-linear persistence trajectories require empirical verification through longitudinal and multi-level research designs. These mechanisms should therefore be interpreted as theoretically grounded propositions that define an agenda for future investigation rather than as established causal relationships.

In summary, Behavioural Alignment Theory advances widening participation research by offering a mechanism-explicit, non-linear and theoretically integrated framework. It incorporates structural and behavioural explanation, specifies how persistence emerges through interaction, and provides a basis for empirical testing and institutional design. In doing so, it contributes both to the refinement of widening participation theory and to the broader development of behavioural science as a discipline concerned with the interaction between individuals and institutional systems.

## 5. Conclusions

This article has addressed a central limitation in widening participation research: the absence of a mechanism-explicit account of how persistence unfolds over time, particularly among widening access mature students. While existing scholarship has provided sophisticated explanations of structural inequality and access, it has been less precise in modelling the behavioural processes through which participation is sustained or disrupted within institutional contexts.

In response, the article has advanced Behavioural Alignment Theory (BAT), defined here as:

*“a mechanism-explicit behavioural science theory that explains mature student persistence as an emergent outcome of institutional–psychological coherence across motivational regulation, expectancy recalibration and instructional architecture”*.

The associated Behavioural Alignment Model (Figure 1) operationalises this theory by illustrating how these three domains interact dynamically to stabilise or destabilise persistence.

The core argument is that persistence cannot be reduced to discrete variables such as motivation, teaching quality or student ability. Rather, as formalised in the Behavioural Alignment Model, it may operate as a non-linear and dynamic process, particularly where role complexity, cost and institutional uncertainty interact. Where pedagogical and organisational conditions support autonomy, scaffold competence, provide clear and consistent signals of expectation, and ensure cognitively manageable learning, these domains align and reinforce one another, producing stable engagement. Conversely, where misalignment occurs through opaque assessment, excessive cognitive demand or pedagogical practices that constrain agency, persistence becomes fragile irrespective of initial motivation or formal access. The model therefore gives analytical form to BAT by specifying how alignment and misalignment unfold over time.

In advancing BAT, this article makes three linked contributions. First, it shifts the study of mature student persistence from lists of predictors to a mechanism-based explanation of how persistence is sustained or weakened over time. Rather than treating motivation, expectancy and instructional architecture as separate variables, BAT explains how they interact: motivation shapes students’ willingness to continue, expectancy shapes whether success still appears possible, and instructional design shapes whether learning remains manageable. Second, it extends sociological and educational accounts by showing how structural disadvantage becomes behaviourally consequential in everyday academic life. Institutional misrecognition, unclear assessment, poor feedback, high perceived cost and excessive cognitive burden influence students’ competence experiences, expectations of success and decisions about continuation. In this way, BAT complements theories of capital, integration and engagement by linking macro-level inequality and institutional conditions to the micro-level processes through which mature students judge whether study remains worthwhile, possible and manageable, without reducing persistence to individual disposition or deficit. Third, it reframes widening participation as a problem of behavioural alignment. Access remains essential, but equitable participation also depends on the design of institutional conditions that sustain motivation, provide credible signals of progress and reduce unnecessary cognitive burden after entry. BAT therefore contributes to behavioural science by treating persistence as a sequence of continuation decisions shaped by institutional environments, rather than as a static outcome or individual trait.

At the same time, both the theory and the model warrant critical reflection. While BAT provides conceptual integration, it introduces risks of complexity, measurement difficulty and potential over-formalisation. The Behavioural Alignment Model, in representing interaction through a non-linear system, may be misinterpreted as implying deterministic or multiplicative relationships, whereas in practice persistence allows for partial compensation and temporal fluctuation. In addition, the framework relies on constructs such as motivation, expectancy and cognitive load that are contextually variable and empirically challenging to operationalise with precision. Furthermore, although BAT resists psychological reductionism, its application requires careful attention to ensure that structural inequality is not inadvertently reframed as a problem of individual adaptation.

Despite these limitations, the framework may have relevance beyond mature students where similar behavioural conditions are present. The conditions under which BAT is most applicable, including role complexity, time constraint, instrumental motivation and educational discontinuity, are no longer confined to mature students but increasingly characterise contemporary higher education systems. As participation becomes more diverse and non-linear, the Behavioural Alignment Model provides a transferable design logic for understanding persistence across post-traditional learning environments.

The article also establishes a foundation for future research. By specifying mechanisms and articulating testable propositions, BAT invites longitudinal, multi-level and mixed-methods inquiry capable of capturing the temporal dynamics of alignment. Future work should examine how institutional signalling shapes expectancy trajectories, how pedagogical relevance supports motivational internalisation, and how instructional design conditions cognitive sustainability under constraint.

## Data Availability

No new data were created or analysed in this study.

## Abbreviations

The following abbreviations are used in this manuscript:

WAMS	Widening Access Mature Students
SDT	Self-Determination Theory
EVT	Expectancy–Value Theory
CLT	Cognitive Load Theory
BAT	Behavioural Alignment Theory
